# Recent Advances in OMICs Technologies and Application for Ensuring Meat Quality, Safety and Authenticity

**DOI:** 10.3390/foods11162532

**Published:** 2022-08-22

**Authors:** Mohammed Gagaoua

**Affiliations:** Food Quality and Sensory Science Department, Teagasc Food Research Centre, Ashtown, D15 KN3K Dublin, Ireland; gmber2001@yahoo.fr or mohammed.gagaoua@teagasc.ie

Consumers and stakeholders are increasingly demanding that the meat industry guarantees high-quality meat products with stable and acceptable sensory and safety properties. To achieve this lofty goal, it is prerequisite for meat researchers to address current meat quality issues and consider certain important goals. First, it is essential to decipher the unknowns concerning the underlying mechanisms of meat quality determination and development. Second, we need a better understanding of the biochemical pathways behind the conversion of muscle into fresh meat and those related to the manufacturing steps and their impact on processed meat products. Third, it is more than necessary to refine our knowledge on the impact of pre- and post-harvest procedures on both the molecular aspects of muscle foods and the final quality and safety of meat products in order to develop management and decision tools. Over the last two decades, sophisticated OMICs technologies—genomics, transcriptomics, proteomics, peptidomics, metabolomics and lipidomics, also known as foodomics—have been powerful approaches that extended the scope of traditional methods and opened up impressive possibilities to explore the above objectives in significant ways [1,2,3,4,5,6]. Foodomics are used for in-depth characterization and better management of numerous food products including for muscle foods. Overall, these techniques aimed to study in a comprehensive manner the dynamic link(s) between the genome and the quality traits of the meat we eat compared to the traditional methods, hence improving both the accuracy and sensitivity thanks to the large quantities of data that can be generated. Accordingly, this Special Issue focused on cutting-edge research applications of OMICs tools to characterize or manage the quality of muscle foods. Eleven published papers applied transcriptomics, targeted and untargeted proteomics, metabolomics, and genomics, among others, to evaluate meat quality, to determine the molecular profiles of meat and meat products, to discover and/or evaluate biomarkers of meat quality traits, and to characterize the safety, the adulteration, and the authenticity of meat and meat products.

In the frame of the discovery and evaluation of beef quality biomarkers, González-Blanco et al. [7] assessed different extraction methods of the sarcoplasmic and myofibrillar sub-proteomes of the *Longissimus thoracis et lumborum* (LTL) to evaluate the most reliable protocol for the identification of biomarkers of dark-cutting beef condition, also known as dark, firm, and dry (DFD) meat [8]. By means of one-dimensional sodium dodecyl-sulfate polyacrylamide gel electrophoresis (SDS-PAGE), the authors investigated the protein fractions of each extraction protocol. Within the sarcoplasmic sub-proteome, the extraction buffers that contain Triton X-100 led to a higher protein extractability, while TES buffer containing Tris, EDTA, and Sucrose was effective to distinguish differences in the protein pattern between the normal and DFD meat. Within the myofibrillar sub-proteome, the non-denaturing buffer allowed higher intensity protein bands while the lysis buffer increased protein extractability with more sensitivity in the differences between the treatments. In a following paper, Sierra et al. [9] focused on the myofibrillar sub-proteome to explore the effects of production systems (intensive versus extensive) and transport and lairage (mixing versus non-mixing with unfamiliar animals) and the post-mortem time ageing (rate and extent of tenderization) of LTL muscle of yearling bulls. Twenty-one proteins were differentially abundant due to any of the factors considered: farm, transport and lairage, and post-mortem time ageing. The proteins were from three major and interconnected pathways, such as muscle structure and associated proteins, energy metabolism and associated pathways, and heat shock proteins, of which several were known biomarkers of beef tenderness [4,10]. The study by Zhu et al. [11] applied a shotgun proteomics approach to identify biomarkers of beef tenderness evaluated using Warner–Bratzler shear force on young Limousin-sired bulls reared under an Irish production system. The authors revealed 34 putative protein biomarkers discriminating between the tender and tough meat groups. These proteins belong to biological pathways related to muscle structure, heat shock proteins, energy metabolism, response to oxidative stress, and apoptosis, from which 23 belong to the previous list of biomarkers of beef tenderness gathered by Gagaoua and co-workers in one repertoire by means of an integromics data mining approach [4]. Furthermore, Zhu et al. proposed a regression model using three proteins (Myozenin 3, Bridging Integrator-1, and Mimecan) that yielded a predictive power of 79%. Another study by Gagaoua et al. [12] aimed to evaluate, by means of Reverse Phase Protein Array (RPPA) quantification (a quantitative microformat Dot-Blot approach), a list of 20 protein biomarkers previously shortlisted to explain and predict both tenderness (evaluated by WBSF) and marbling (intramuscular fat (IMF) content) of 188 Protected Designation of Origin (PDO) Maine-Anjou cows. Using three statistical methods, namely, correlations analyses, clustering of WBSF and marbling into three quality clusters, and Partial Least Squares regressions (PLS-R), several biomarkers were selected. Whatever the statistical method, seven putative biomarkers for both WBSF values and marbling were qualified as being robust, hence allowing the authors to move forward in the pipeline of biomarker discovery for beef eating qualities. In this study, 10 and 9 proteins were qualified using a large database as significantly related to the determination of beef tenderness and marbling, respectively, in PDO Maine-Anjou cows.

In lamb research, two papers evaluated the variation in color [13] using proteomics and tenderness using a combination of Iso-seq, RNA-seq, and CTCF ChIP-seq data [14]. The first study by Gao et al. [13] investigated the sarcoplasmic and myrofibrillar sub-proteomes of *Longissimus lumborum* (LL, color-stable) and *Psoas major* (PM, colour-labile) from Small-tailed Han sheep in relation to color stability during post-mortem storage (1, 3, and 5 days). The study revealed that the main differentially abundant proteins were from the glycolysis, others belong to the energy metabolism enzymes, chaperones and heat shock proteins, and proteins of structure. Thanks to correlation analyses, proteins such as adenylate kinase isoenzyme 1 (AK1), Pyruvate kinase (PKM), Carbonic anhydrase 3 (CA3), and Creatine kinase M-type (CKM) were significantly related to color stability in agreement with the available proteome repertoire of meat color [5]. This study allowed to validate predictors of color discoloration in sheep meat during storage. The second paper (a communication) by Yuan et al. [14] performed an experiment on sheep from two crossbred populations, Dorper x Hu x Hu (DHH) and Dorper x Dorper x Hu (DDH), with divergent meat tenderness. The authors aimed to identify key isoforms associated with this important quality and to better understand the underlying mechanisms of alternative splicing regulations leading to the production of isoforms. The authors revealed in this preliminary study 624 differentially expressed isoforms between DDH and DHH.

Meat and processed meat products have high nutritional value and economic importance, which makes them appealing commodities for fraudulent activities. Fraud activities associated with meat and meat products include addition (allergic proteins, preservatives), dilution (addition of water for yield increase and cost reduction), substitution, and mislabeling or misdescription, which are critical issues for economic, health, and religious reasons. Therefore, meat authentication is an important concern to protect consumers from illegal and unwanted ingredients. Accordingly, three papers dealing with meat authenticity, origin, and detection of meat adulteration using OMICs methods were published [15,16,17]. Cai et al. [15] proposed a simple and reliable single-tube septuple PCR assay based on mitochondrial DNA to simultaneously recognize seven meat species from pig, beef, sheep, chicken, turkey, goose, and duck. Furthermore, the authors validated the method in terms of sensitivity, specificity, robustness, and low costs for broad application to detect the origin of meat in foodstuffs with suspected adulteration. Another interesting study by Dobrovolny et al. [16] consisted of a collaborative work among 15 laboratories (inter-laboratory ring trial) that aimed to harmonize an analytical method based on DNA metabarcoding assay to detect adulteration from poultry and mammalian species. In this European study, each research team received and analyzed 16 anonymously labeled samples (8 samples, 2 subsamples each) containing six mixtures of DNA extract, one DNA extract from a model sausage, and another from maize, considered in this trial as a negative control. The evaluation parameters of the method allowed the researchers to confirm the reliability of the DNA metabarcoding approach for meat species authentication in routine analysis. The third study by Chen et al. [17] developed a duck genomic reference material by means of digital PCR platforms to detect meat adulteration through the detection of the duck *interleukin 2* (*IL2*) gene. Similarly, eight independent laboratories proceeded with the validation and certification of the proposed method

Two other research papers aiming to evaluate the freshness in gilthead sea bream (*Sparus aurata*) using metabolomics [18] and a better understanding of wooden breast myopathy in commercial broilers using proteomics [19] were published in this Special Issue.

In summary, the content of this Special Issue fits in with the current trend toward the use of foodomics to ensuring the quality, safety, and authenticity of meat and meat products. We hope that this Special Issue will attract the interest of the community of meat scientists, as well as students and scholars, by inspiring them to undertake more research in this emerging and important area of research towards the development of methods and decision tools to ensure more sustainable muscle foods. Special thanks go to the authors for their valuable contributions to this Special Issue and to our colleagues who devoted their time to review the papers. We sincerely hope that readers will find this Special Issue on meat OMICS-based approaches motivating and informative.
